# Supplementary material for: The N6-methyladenosine methyltransferase METTL16 enables erythropoiesis through safeguarding genome integrity
Source: Nat Commun. 2022 Oct 28;13:6435. doi: 10.1038/s41467-022-34078-y (PMC9616860; doi:10.1038/s41467-022-34078-y)
Supplement: Supplementary file 1 — Supplementary Information [file 41467_2022_34078_MOESM1_ESM.pdf]

# Supplementary information

# The $N^6$-methyladenosine methyltransferase METTL16 enables erythropoiesis through safeguarding genome integrity

Masanori Yoshinaga[1,*], Kyuho Han[2], David W Morgens[2], Takuro Horii[3], Ryosuke Kobayashi[3], Tatsuaki Tsuruyama[4], Fabian Hia[1], Shota Yasukura[1], Asako Kajiya[1], Ting Cai[1], Pedro HC Cruz[5], Alexis Vandenbon[6], Yutaka Suzuki[7], Yukio Kawahara[5], Izuho Hatada[3,8], Michael C Bassik[2], and Osamu Takeuchi[1,*]

[1]Department of Medical Chemistry, Graduate School of Medicine, Kyoto University, Kyoto 606-8501, Japan

[2]Department of Genetics, Stanford University School of Medicine, California 94305, USA

[3]Laboratory of Genome Science, Biosignal Genome Resource Center, Institute for Molecular and Cellular Regulation, Gunma University, Gunma 371-8512, Japan

[4]Department of Drug and Discovery Medicine, Graduate School of Medicine, Kyoto University, Kyoto 606-8501, Japan

[5]Department of RNA Biology and Neuroscience, Graduate School of Medicine, Osaka University, Osaka 565-0871, Japan

[6]Laboratory of Systems Virology, Department of Virus Research, Institute for Frontier Life and Medical Sciences, Kyoto University, Kyoto 606-8507, Japan

[7]Laboratory of Functional Genomics, Department of Medical Genome Sciences, Graduate School of Frontier Sciences, The University of Tokyo, Chiba 277-8562, Japan

[8]Viral Vector Core, Gunma University Initiative for Advanced Research (GIAR), Gunma 371-8512, Japan

*Correspondence: otake@mfour.med.kyoto-u.ac.jp (O.T.) and m_yoshi@mfour.med.kyoto-u.ac.jp (M.Y.)

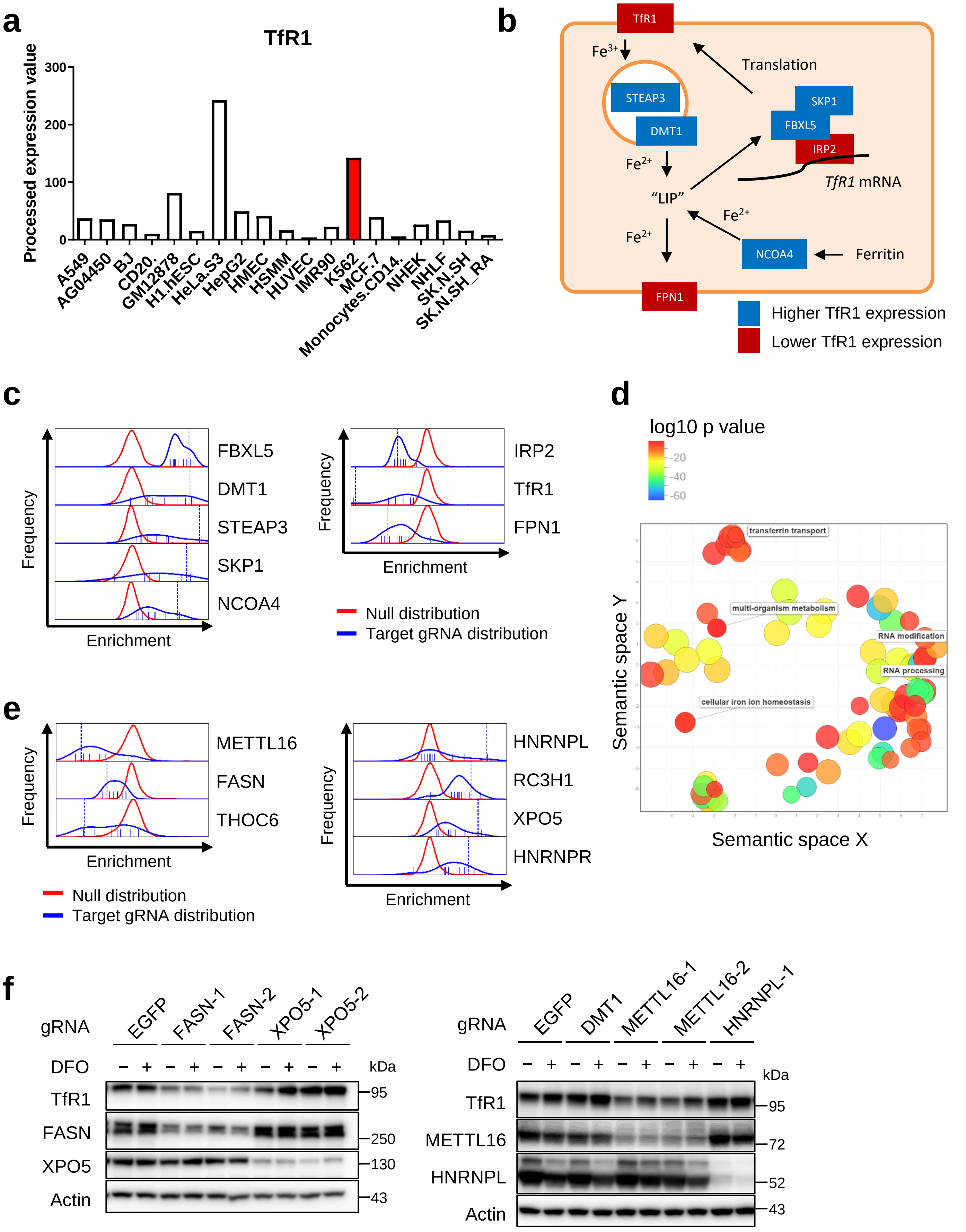
a
TfR1
Processed expression value
300
200
100
0
A549
AG04450
BJ
CD20.
GM12878
H1.hESC
HeLa.S3
HepG2
HMEC
HSMM
HUVEC
IMR90
K562
MCF.7
Monocytes.CD14.
NHEK
NHLF
SK.N.SH
SK.N.SH_RA
b
TfR1
Fe3+
Translation
STEAP3
DMT1
SKP1
FBXL5
IRP2
Fe2+
TfR1 mRNA
"LIP"
Fe2+
Fe2+
NCOA4
Ferritin
FPN1
Higher TfR1 expression
Lower TfR1 expression
c
Frequency
FBXL5
DMT1
STEAP3
SKP1
NCOA4
Enrichment
Frequency
IRP2
TfR1
FPN1
Enrichment
Null distribution
Target gRNA distribution
d
log10 p value
-20
-40
-60
transferrin transport
multi-organism metabolism
RNA modification
RNA processing
cellular iron ion homeostasis
Semantic space Y
Semantic space X
e
Frequency
METTL16
FASN
THOC6
Enrichment
Null distribution
Target gRNA distribution
Frequency
HNRNPL
RC3H1
XPO5
HNRNPR
Enrichment
f
gRNA
EGFP
FASN-1
FASN-2
XPO5-1
XPO5-2
DFO
− + − + − + − + − +
kDa
TfR1
FASN
XPO5
Actin
95
250
130
43
gRNA
EGFP
DMT1
METTL16-1
METTL16-2
HNRNPL-1
DFO
− + − + − + − + − +
kDa
TfR1
METTL16
HNRNPL
Actin
95
72
52
43

**Supplementary Fig. 1: Validation of CRISPR screen results confirms multiple RNA binding proteins which regulate TfR1 expression.**

(a) *TfR1* mRNA expression levels in various cells from ENCODE database.

(b) Schematic representation of the TfR1 CRISPR screen hits. The genes related to iron metabolism were shown.

(c) Enrichment of gRNAs and estimated effect size for known iron regulatory genes.

(d) GO analysis of the CRISPR screen top hits by REVIGO[1].

(e) Enrichment of gRNAs and estimated effect size for novel RBPs regulating TfR1 expression.

(f) Effects of the genetic ablation of DMT1, FASN, XPO5, METTL16 and HNRNPL on TfR1 protein expression. K562-Cas9 cells were transduced with lentivirus vectors expressing indicated gRNAs and cultured in the presence or absence of an iron chelator, deferoxamine mesylate (DFO) overnight. DFO treatment minimizes the effect of the change in intracellular iron concentration, which indirectly influences TfR1 expression. Similar results were obtained in two independent experiments.

Red lines represent the distribution of non-targeting controls. Blue vertical lines represent the enrichment of individual gRNAs targeting indicated genes. Blue curved lines represent the smoothed distribution of the enrichment of gRNAs targeting the indicated genes. Blue vertical dotted lines represent the effect size estimated by casTLE (c, e). Source data are provided as a Source Data file.

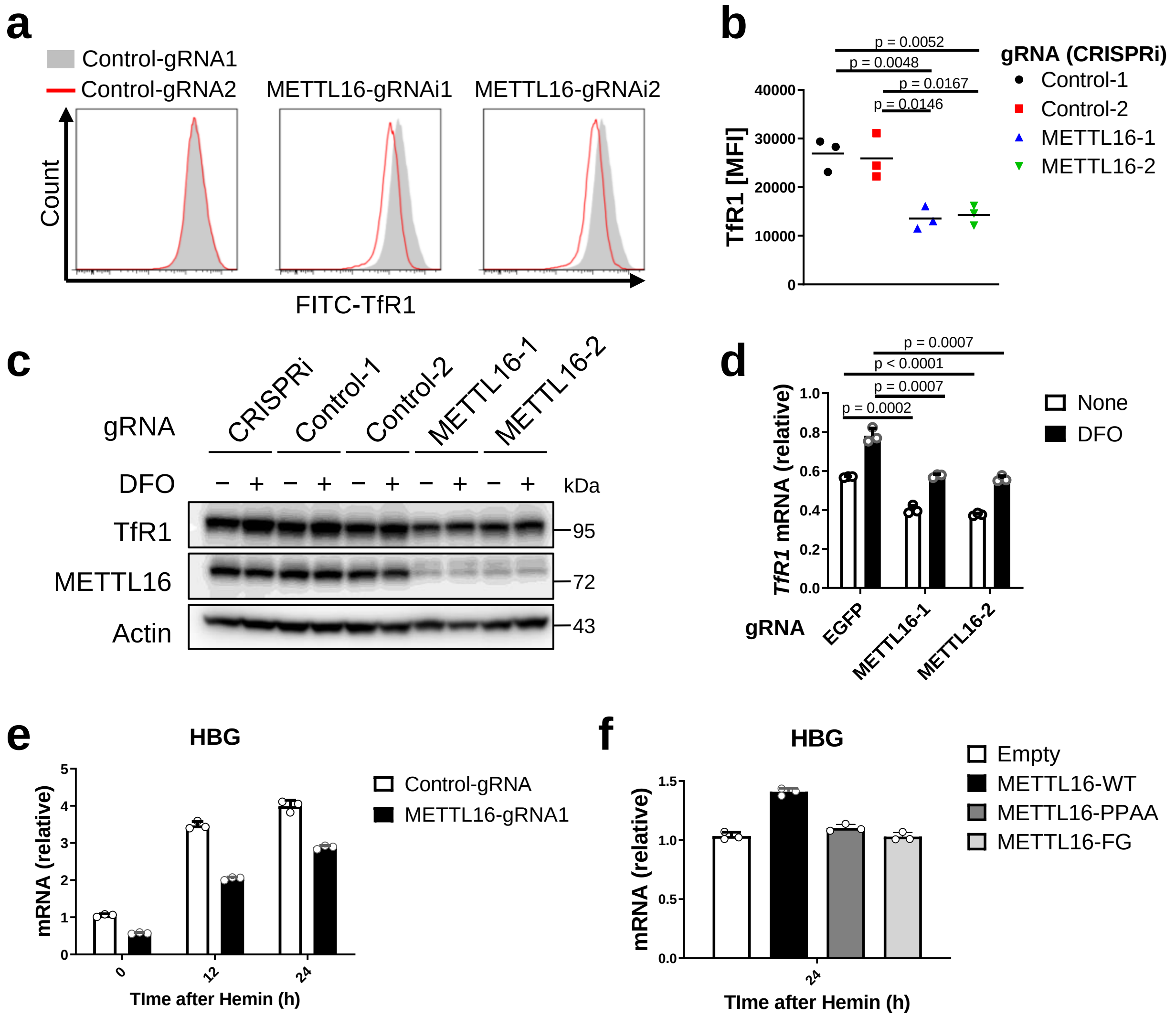


**Supplementary Fig. 2: METTL16 regulates TfR1 expression in a manner dependent on its methyltransferase activity.**

(a-b) Surface TfR1 expression under METTL16 knockdown by CRISPRi in the presence of DFO (a). TfR1 mean fluorescence intensity (MFI) in each population (n=3 independent experiments, b). Horizontal lines indicate the mean.

(c) Effects of METTL16 knockdown by CRISPRi on TfR1 protein expression. K562-CRISPRi cells were transduced with lentivirus vectors expressing indicated gRNAs and cultured in the presence or absence of DFO overnight. Similar results were obtained in at least two independent experiments.

(d) *TfR1* mRNA expression in K562-Cas9 cells lacking METTL16 in the presence and absence of DFO (n=3 technical replicates).

(e) *HBG* mRNA expression in K562-Cas9 cells lacking METTL16 upon hemin treatment (n=3 technical replicates).

(f) *HBG* mRNA expression in METTL16-gRNA1 expressing K562-Cas9 cells reconstituted with wild-type or mutant METTL16 upon hemin treatment (n=3 technical replicates).

Data are expressed as mean ± SD (d-f). The p values were calculated using two-tailed Student's t test (b, d). Source data are provided as a Source Data file.

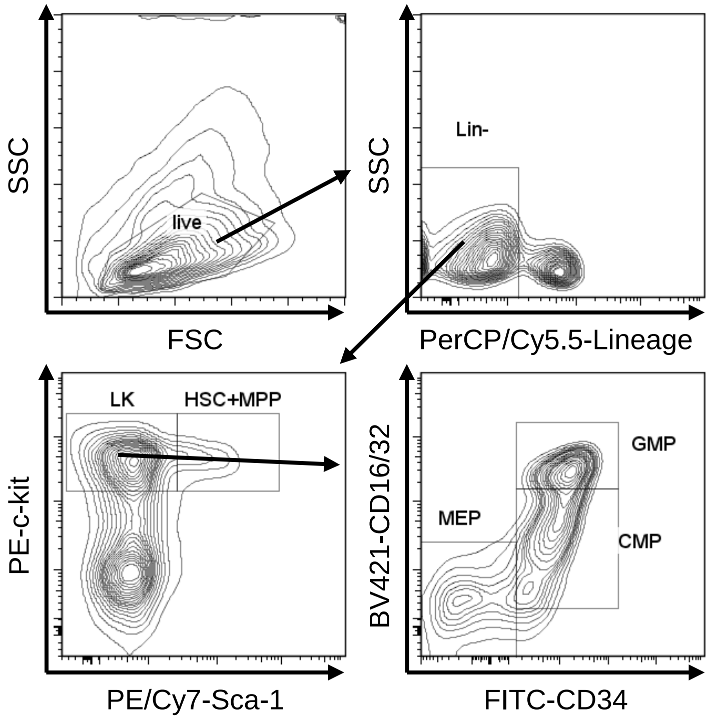


**Supplementary Fig. 3: Gating strategy for sorting hematopoietic stem/progenitor cells in the bone marrow.**

The representative gating strategy for isolating hematopoietic stem and progenitor cells in the bone marrow in Fig. 2a. Lineage-negative cells were magnetically sorted from total bone marrow cells, then stained with indicated antibodies and analyzed by flow cytometry.

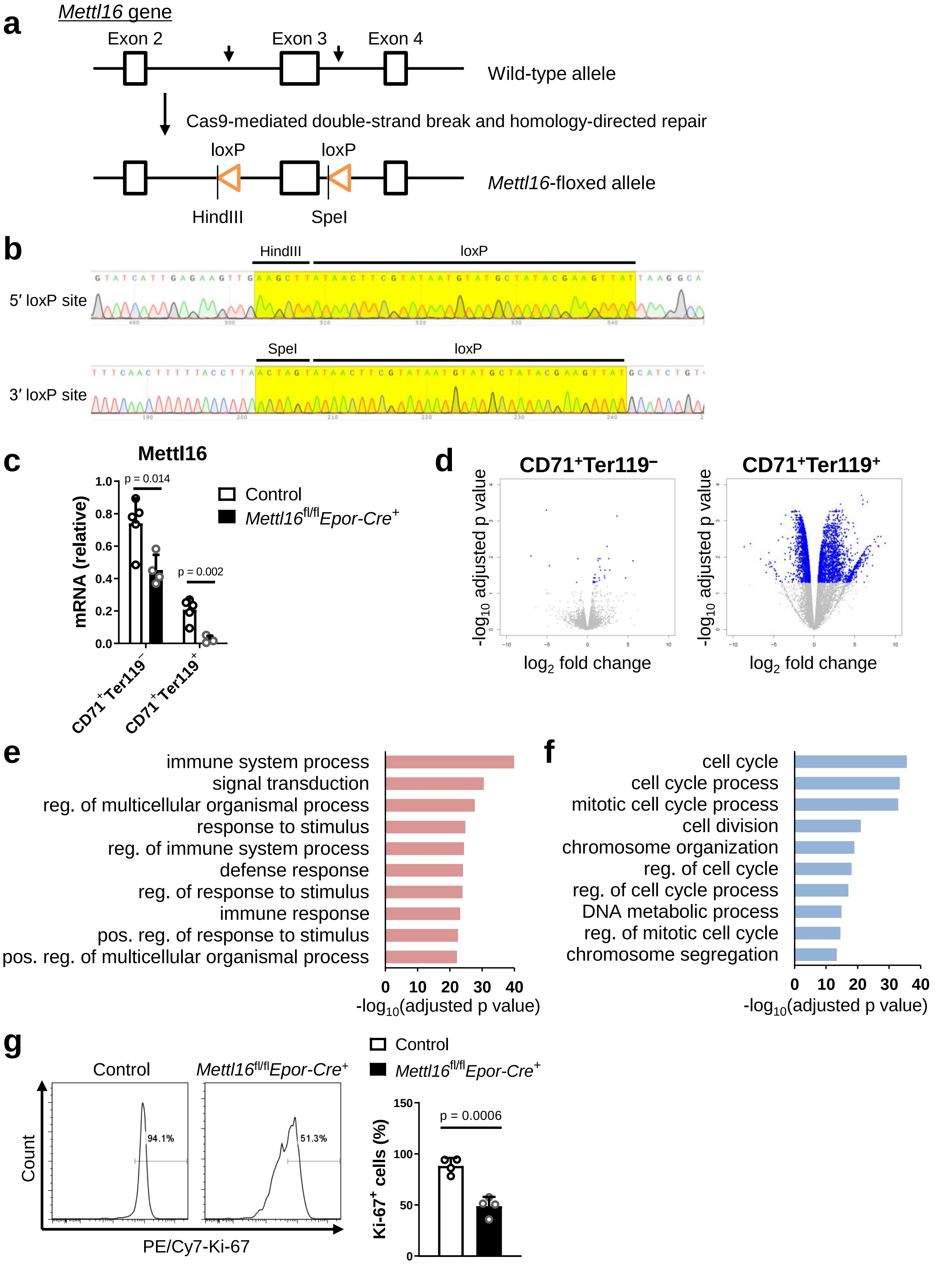
a
Mettl16 gene
Exon 2
Exon 3
Exon 4
Wild-type allele
Cas9-mediated double-strand break and homology-directed repair
loxP
loxP
Mettl16-floxed allele
HindIII
SpeI
b
HindIII
loxP
5′ loxP site
SpeI
loxP
3′ loxP site
c
Mettl16
mRNA (relative)
p = 0.014
p = 0.002
Control
Mettl16fl/flEpor-Cre+
CD71+Ter119–
CD71+Ter119+
d
CD71+Ter119–
CD71+Ter119+
-log10 adjusted p value
log2 fold change
e
immune system process
signal transduction
reg. of multicellular organismal process
response to stimulus
reg. of immune system process
defense response
reg. of response to stimulus
immune response
pos. reg. of response to stimulus
pos. reg. of multicellular organismal process
-log10(adjusted p value)
f
cell cycle
cell cycle process
mitotic cell cycle process
cell division
chromosome organization
reg. of cell cycle
reg. of cell cycle process
DNA metabolic process
reg. of mitotic cell cycle
chromosome segregation
-log10(adjusted p value)
g
Control
Mettl16fl/flEpor-Cre+
94.1%
51.3%
Count
PE/Cy7-Ki-67
Control
Mettl16fl/flEpor-Cre+
p = 0.0006
Ki-67+ cells (%)

**Supplementary Fig. 4: Generation and phenotypic analysis of *Mettl16*$^{fl/fl}$*Epor-Cre*$^{+}$ mice.**

(a) Schematic of experimental procedures to generate *Mettl16*-floxed allele via CRISPR-mediated genome editing.

(b) Raw electropherograms of generated *Mettl16*-floxed allele from the founder mouse.

(c) *Mettl16* mRNA expression levels in CD71$^{+}$Ter119$^{–}$ and CD71$^{+}$Ter119$^{+}$ erythroblasts from control (n=5 mice) and *Mettl16*$^{fl/fl}$*Epor-Cre*$^{+}$ mice (n=4 mice).

(d) Volcano plot showing transcriptome alteration between CD71$^{+}$Ter119$^{–}$ and CD71$^{+}$Ter119$^{+}$ erythroblasts from control and *Mettl16*$^{fl/fl}$*Epor-Cre*$^{+}$ mice. Blue dots represent differentially regulated genes. Statistical analysis was performed using limma.

(e-f) GO analysis of upregulated (e) and downregulated (f) mRNAs in erythroblasts from *Mettl16*$^{fl/fl}$*Epor-Cre*$^{+}$ mice. Statistical analysis was performed using Immuno-Navigator.

(g) The histogram of Ki-67 intensity in each population (left). The right panel shows the Ki-67 mean fluorescence intensity (MFI) in each population (n=4 mice).

Data are expressed as mean ± SD (c, g). The p values were calculated using two-tailed Student’s t test (c, g). Source data are provided as a Source Data file.

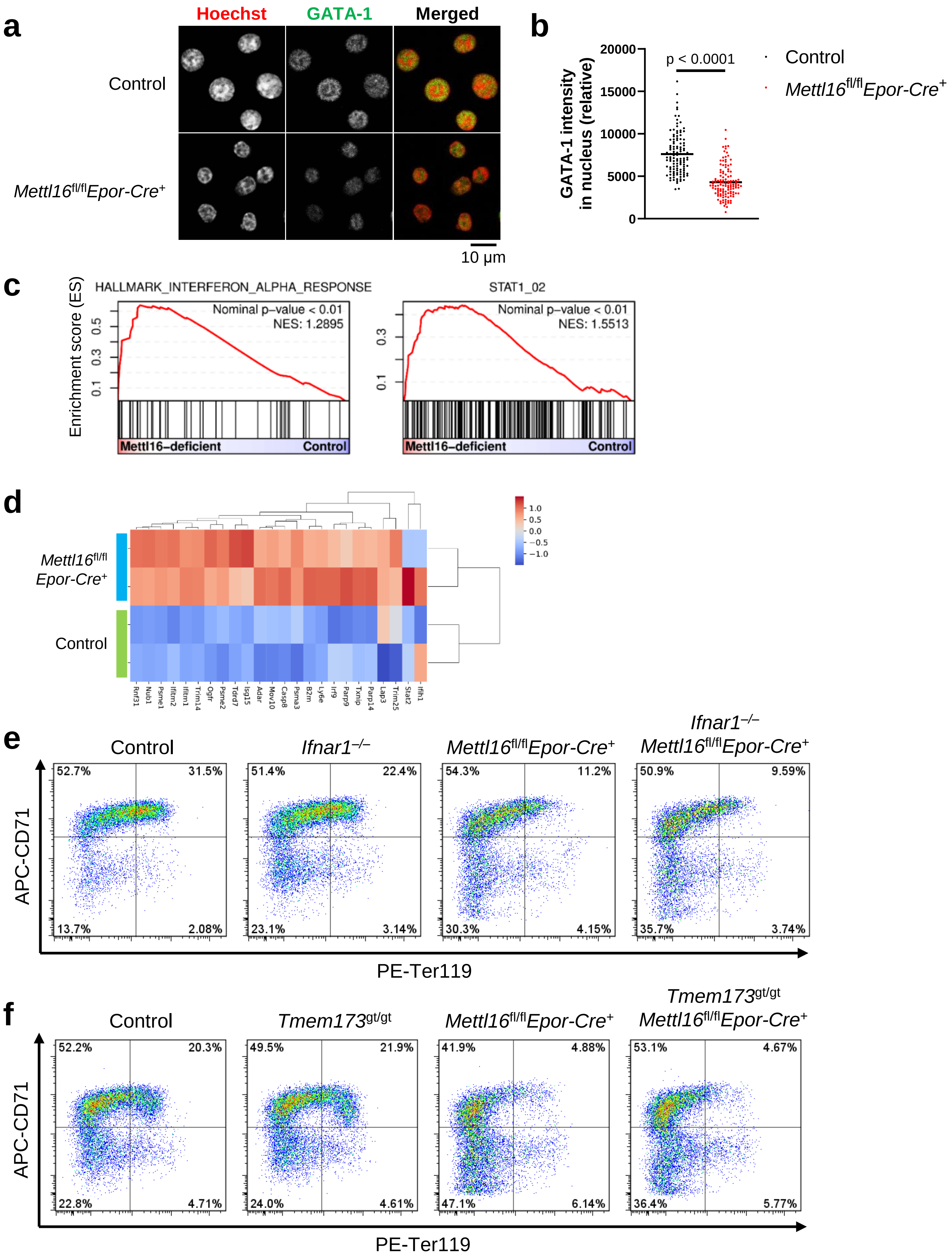
a
Hoechst
GATA-1
Merged
Control
Mettl16fl/flEpor-Cre+
10 μm
b
GATA-1 intensity in nucleus (relative)
p < 0.0001
Control
Mettl16fl/flEpor-Cre+
c
Enrichment score (ES)
HALLMARK_INTERFERON_ALPHA_RESPONSE
Nominal p–value < 0.01
NES: 1.2895
Mettl16–deficient
Control
STAT1_02
Nominal p–value < 0.01
NES: 1.5513
Mettl16–deficient
Control
d
Mettl16fl/fl Epor-Cre+
Control
Rnf31 Nub1 Psme1 Ifitm2 Ifitm1 Trim14 Ogfr Psme2 Tdrd7 Isg15 Adar Mov10 Casp8 Psma3 B2m Ly6e Irf9 Parp9 Txnip Parp14 Lap3 Trim25 Stat2 Ifih1
e
Control
Ifnar1–/–
Mettl16fl/flEpor-Cre+
Ifnar1–/– Mettl16fl/flEpor-Cre+
APC-CD71
PE-Ter119
52.7% 31.5% 13.7% 2.08%
51.4% 22.4% 23.1% 3.14%
54.3% 11.2% 30.3% 4.15%
50.9% 9.59% 35.7% 3.74%
f
Control
Tmem173gt/gt
Mettl16fl/flEpor-Cre+
Tmem173gt/gt Mettl16fl/flEpor-Cre+
APC-CD71
PE-Ter119
52.2% 20.3% 22.8% 4.71%
49.5% 21.9% 24.0% 4.61%
41.9% 4.88% 47.1% 6.14%
53.1% 4.67% 36.4% 5.77%

**Supplementary Fig. 5: METTL16 regulates erythropoiesis via GATA-1, not via STING signaling.**

(a-b) GATA-1 protein expression in $CD71^{+}Ter119^{+}$ erythroblasts of E11.5 fetal liver from control and *Mettl16*$^{fl/fl}$*Epor-Cre*$^{+}$ mice. Erythroblasts were isolated by cell sorting, stained by Hoechst 33342 (red) and GATA-1 antibody (green) and analyzed by the confocal microscopy (a). The quantification of GATA-1 intensity in each cell (b, n=118-122 cells). Horizontal lines indicate the mean. The p values were calculated using two-tailed Student's t test.

(c) GSEA plot showing the enrichment of the hallmarks of interferon alpha response and STAT1 transcriptional targets in erythroblasts from *Mettl16*$^{fl/fl}$*Epor-Cre*$^{+}$ mice. Statistical analysis was performed using the GSEA software.

(d) Heatmap showing the relative mRNA expression levels of interferon-stimulated genes listed in the gene set "the hallmarks of interferon alpha response" in erythroblasts from control and *Mettl16*$^{fl/fl}$*Epor-Cre*$^{+}$ mice.

(e) Flow cytometric analysis of CD71 and Ter119 expression in the E11.5 fetal liver from control, *Ifnar1*$^{-/-}$, *Mettl16*$^{fl/fl}$*Epor-Cre*$^{+}$, and *Ifnar1*$^{-/-}$*Mettl16*$^{fl/fl}$*Epor-Cre*$^{+}$ mice.

(f) Flow cytometric analysis of CD71 and Ter119 expression in the E11.5 fetal liver from control, *Tmem173*$^{gt/gt}$, *Mettl16*$^{fl/fl}$*Epor-Cre*$^{+}$, and *Tmem173*$^{gt/gt}$*Mettl16*$^{fl/fl}$*Epor-Cre*$^{+}$ mice.

Data are representative of at least two independent experiments (a, b, e, and f). Source data are provided as a Source Data file.

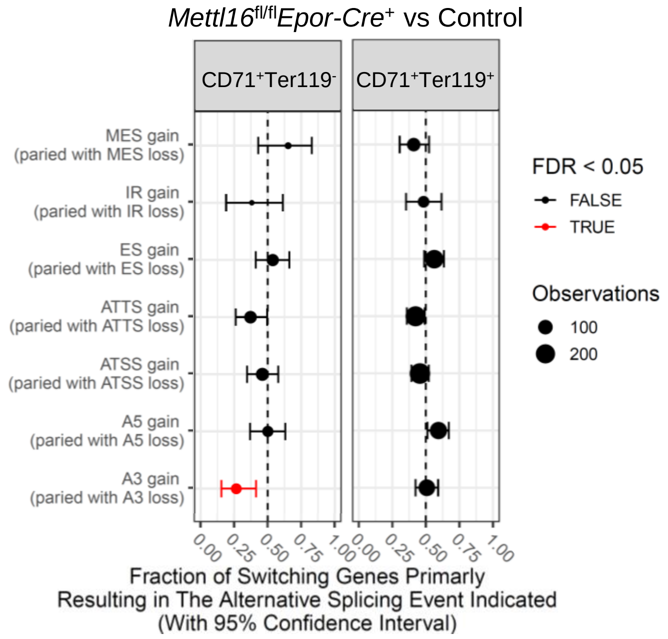


**Supplementary Fig. 6: Alternative splicing analysis in erythroblasts under METTL16 deficiency.** Alternative splicing analysis using the RNA-seq dataset from erythroblasts from control and *Mettl16*$^{fl/fl}$*Epor-Cre*$^{+}$ mice (n=2 biological replicates) by IsoformSwitchAnalyzeR[2]. MES, multiple exon skipping; IR, intron retention; ES, exon skipping; ATTS, alternative transcription termination sites; ATSS, alternative transcription start sites; A5, alternative 5′ donor sites; A3, alternative 3′ acceptor sites. Data are expressed as mean ± 95% confidence interval.

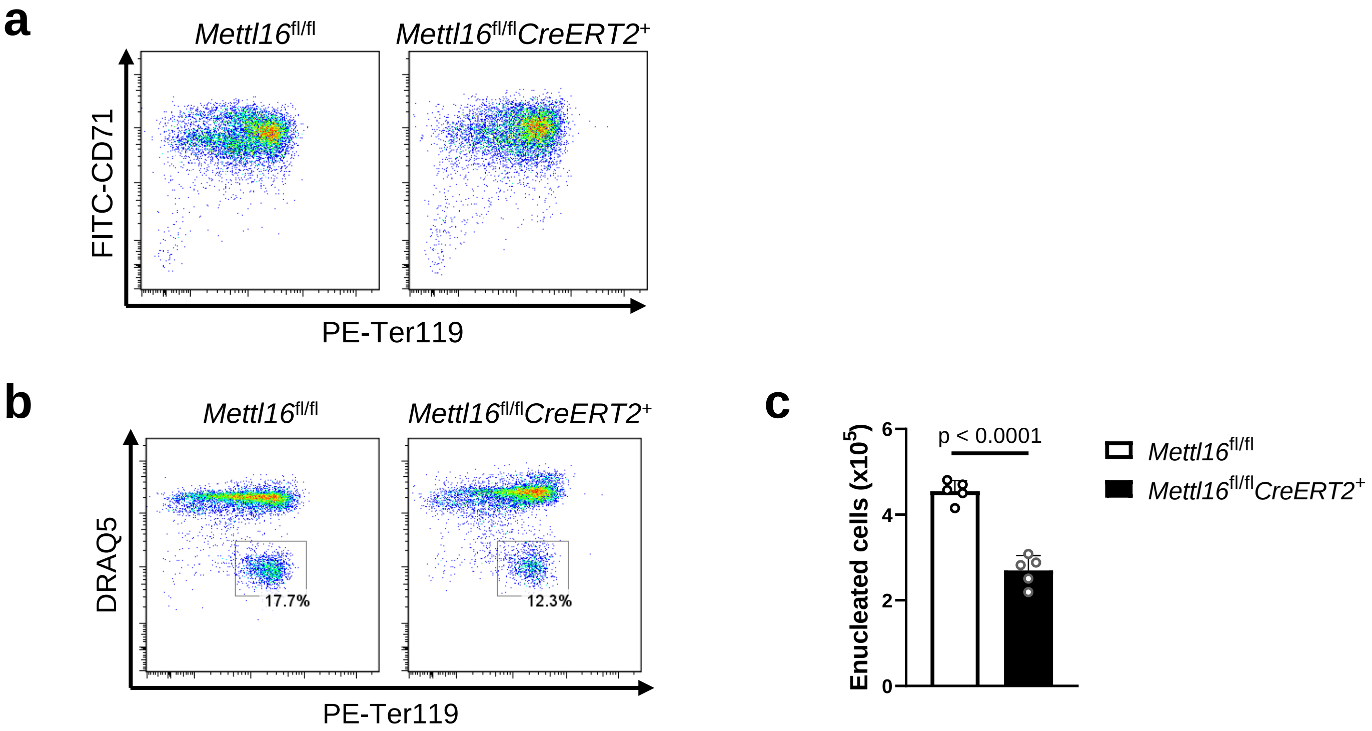


**Supplementary Fig. 7: Phenotypic characterization of METTL16-deficient cultured erythroblasts.**

(a-b) Flow cytometric analysis of the cultured erythroblasts from control and *Mettl16*$^{fl/fl}$*CreERT2*$^{+}$ mice. Lineage-negative fetal liver cells on E13.5 were cultured in Epo and 4-OHT-containing medium and then harvested, stained with indicated antibodies or the nuclear dye DRAQ5, and analyzed using flow cytometry. DRAQ5 negative cells represent enucleated cells.

(c) Enucleated cell numbers in the cultured erythroblasts from E13.5 control and *Mettl16*$^{fl/fl}$*CreERT2*$^{+}$ mice were enumerated (n=5 biological replicates).

Data are expressed as mean ± SD (c). The p values were calculated using two-tailed Student's t test (c). Similar results were obtained in at least two independent experiments. Source data are provided as a Source Data file.

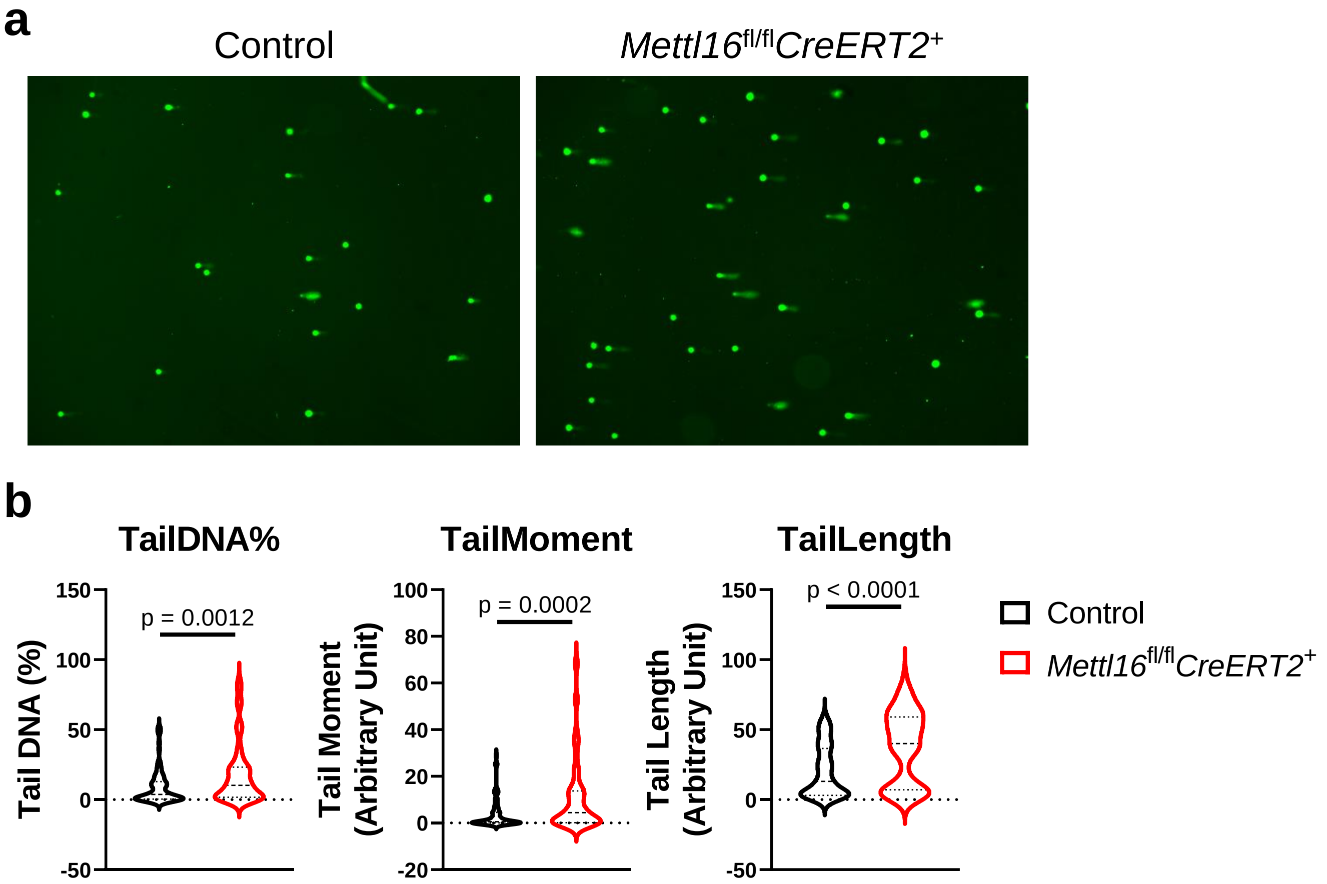


**Supplementary Fig. 8: DNA damage is increased in METTL16-deficient erythroblasts.**

(a-b) Comet assay using the cultured erythroblasts from control and *Mettl16*$^{fl/fl}$*CreERT2*$^{+}$ mice. Lineage-negative fetal liver cells were cultured in Epo and 4-OHT-containing medium, harvested, and subjected to the alkaline comet assay. Representative comet images from each genotype were shown (a).

(b) Comet quantification using CASPLab (n=78 cells for control, n=75 for *Mettl16*$^{fl/fl}$*CreERT2*$^{+}$). DNA content in the tail, the tail moment, and the tail length of each comet were assessed.

The p values were calculated using two-tailed Student's t test. Data are representative of at least two independent experiments (a, b). Source data are provided as a Source Data file.

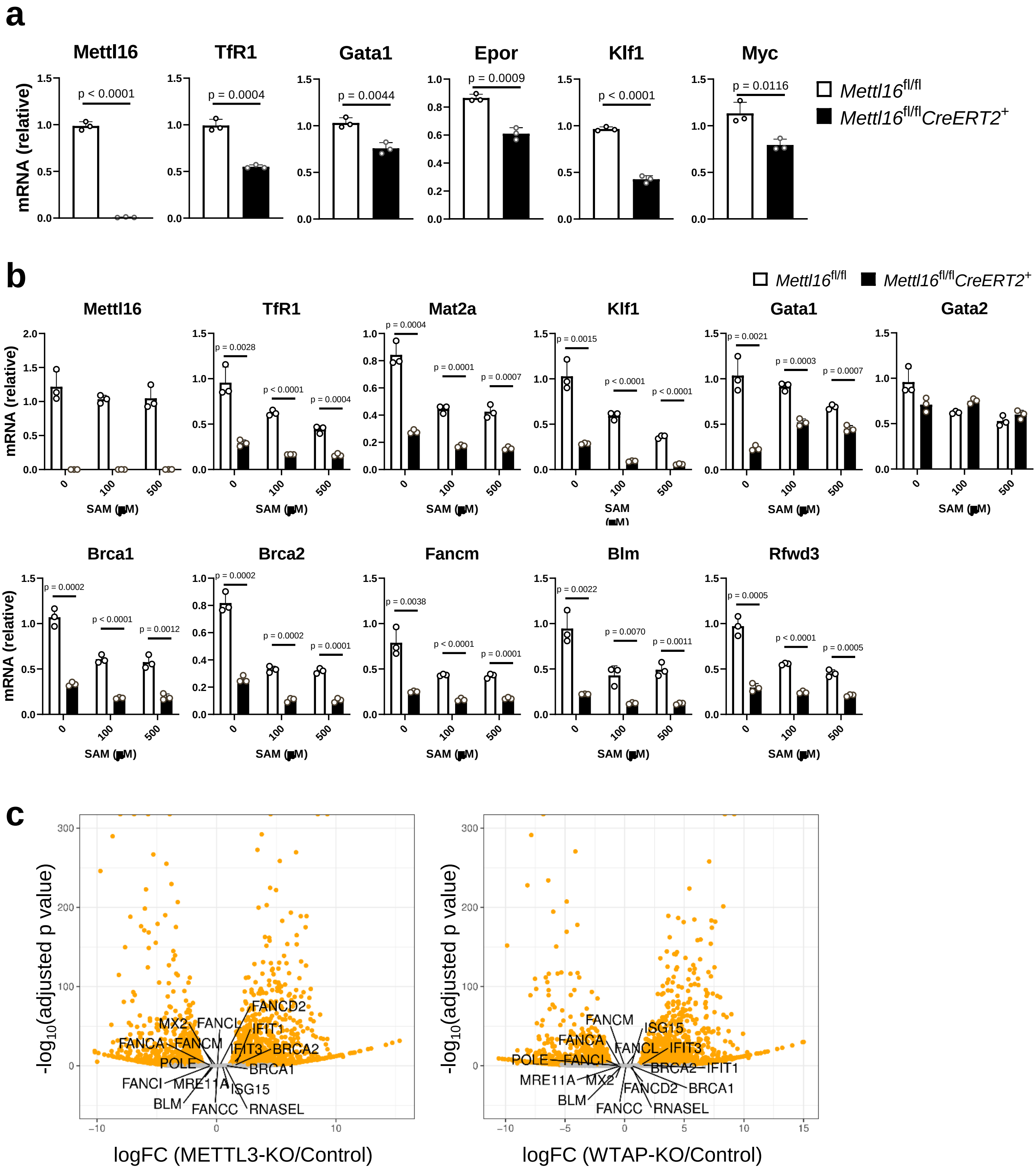

a
Mettl16
TfR1
Gata1
Epor
Klf1
Myc
mRNA (relative)
p < 0.0001
p = 0.0004
p = 0.0044
p = 0.0009
p < 0.0001
p = 0.0116
Mettl16fl/fl
Mettl16fl/flCreERT2+
b
Mettl16
TfR1
Mat2a
Klf1
Gata1
Gata2
Brca1
Brca2
Fancm
Blm
Rfwd3
SAM (μM)
c
logFC (METTL3-KO/Control)
logFC (WTAP-KO/Control)
-log10(adjusted p value)
FANCD2
MX2
FANCL
IFIT1
FANCA
FANCM
IFIT3
BRCA2
POLE
BRCA1
FANCI
MRE11A
ISG15
BLM
FANCC
RNASEL

**Supplementary Fig. 9: METTL16 regulates erythroid gene expression in a MAT2A-independent manner.**

(a) mRNA expression levels in cultured erythroblasts from control and *Mettl16*$^{fl/fl}$*CreERT2*$^{+}$ mice (n=3 biological replicates).

(b) mRNA expression levels in cultured erythroblasts from control and *Mettl16*$^{fl/fl}$*CreERT2*$^{+}$ mice treated with indicated concentrations of S-adenosyl methionine (SAM, n=3 biological replicates).

(c) Volcano plot showing transcriptome alteration between control and METTL3- or WTAP-deficient cells. Datasets generated in the published RNA-seq analysis[3] were reanalyzed. Orange dots represent differentially expressed genes. Adjusted p values were calculated in the original study.

Data are expressed as mean ± SD (a, b). The p values were calculated using two-tailed Student's t test (a, b). Source data are provided as a Source Data file.

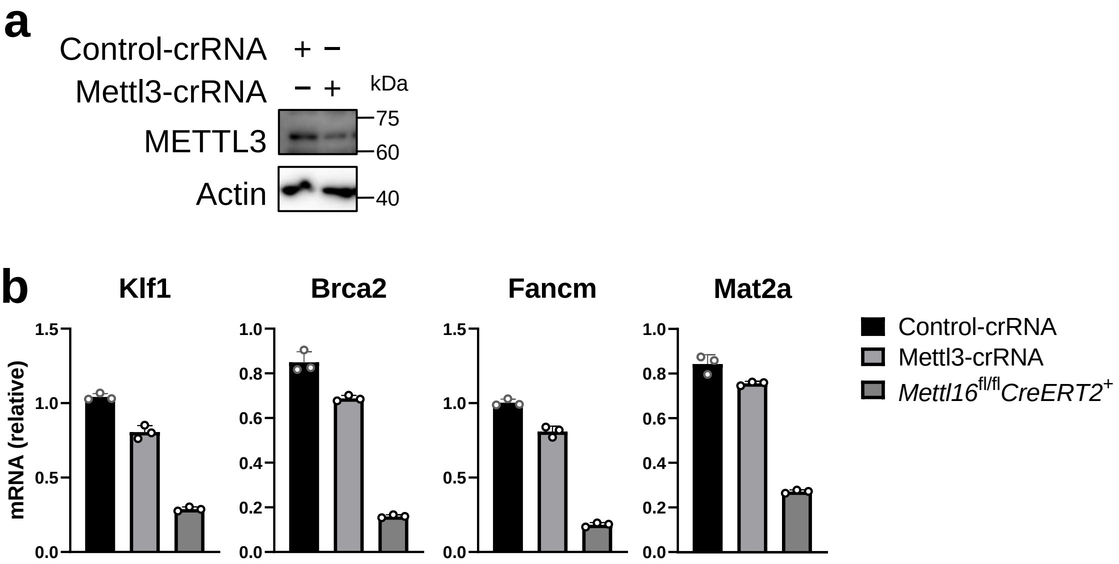


**Supplementary Fig. 10: Effect of METTL3 deficiency in cultured erythroblasts**

(a) Effects of the nucleofection of Cas9 and the crRNA targeting METTL3.

(b) mRNA expression levels of indicated genes in the cultured erythroblasts which were nucleofected with indicated crRNAs targeting METTL3 and Cas9 complexes (n=3 technical replicates). The cultured erythroblasts from *Mettl16*$^{fl/fl}$*CreERT2*$^{+}$ mice which were nucleofected with the control crRNA and Cas9 complex were analyzed together.

Data are expressed as mean ± SD (b). Data are representative of at least two independent experiments. Source data are provided as a Source Data file.

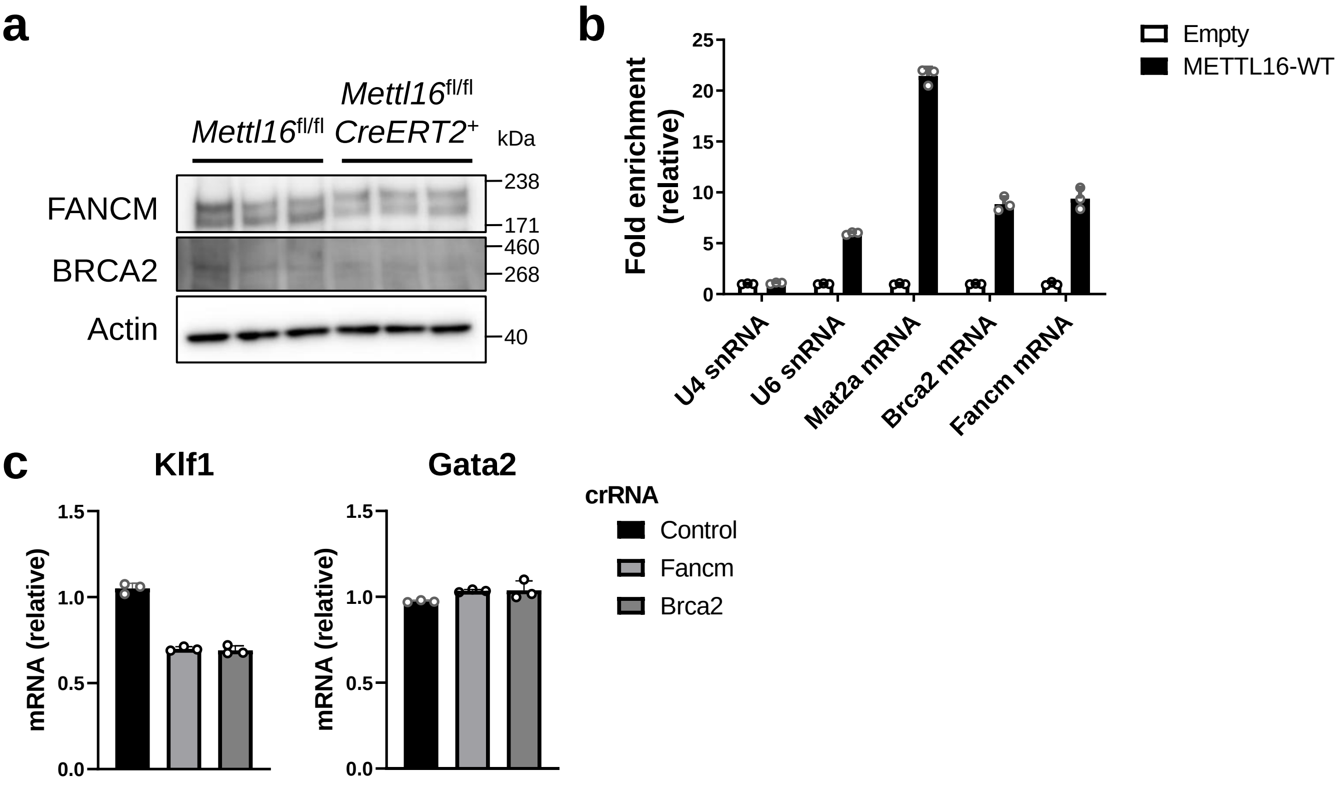


**Supplementary Fig. 11: Additional data regarding the METTL16 targets *Brca2* and *Fancm***

(a) Protein expression levels in cultured erythroblasts from control and *Mettl16*$^{fl/fl}$*CreERT2*$^{+}$ mice (n=3 biologically independent samples).

(b) Enrichment of indicated mRNAs to wild-type METTL16. NIH3T3 cells were transfected with wild-type METTL16 expression vector or the control (Empty). RNA-protein complexes were immunoprecipitated and the levels of U4 and U6 snRNAs, *Mat2a*, *Brca2* and *Fancm* mRNAs were measured by RT-qPCR (n=3 technical replicates). U4 snRNA is a negative control that does not interact with METTL16.

(c) mRNA expression levels of indicated genes in the cultured erythroblasts from wild-type mice which were nucleofected with indicated crRNAs and Cas9 complexes (n=3 technical replicates).

Data are expressed as mean ± SD (b, c). Source data are provided as a Source Data file.

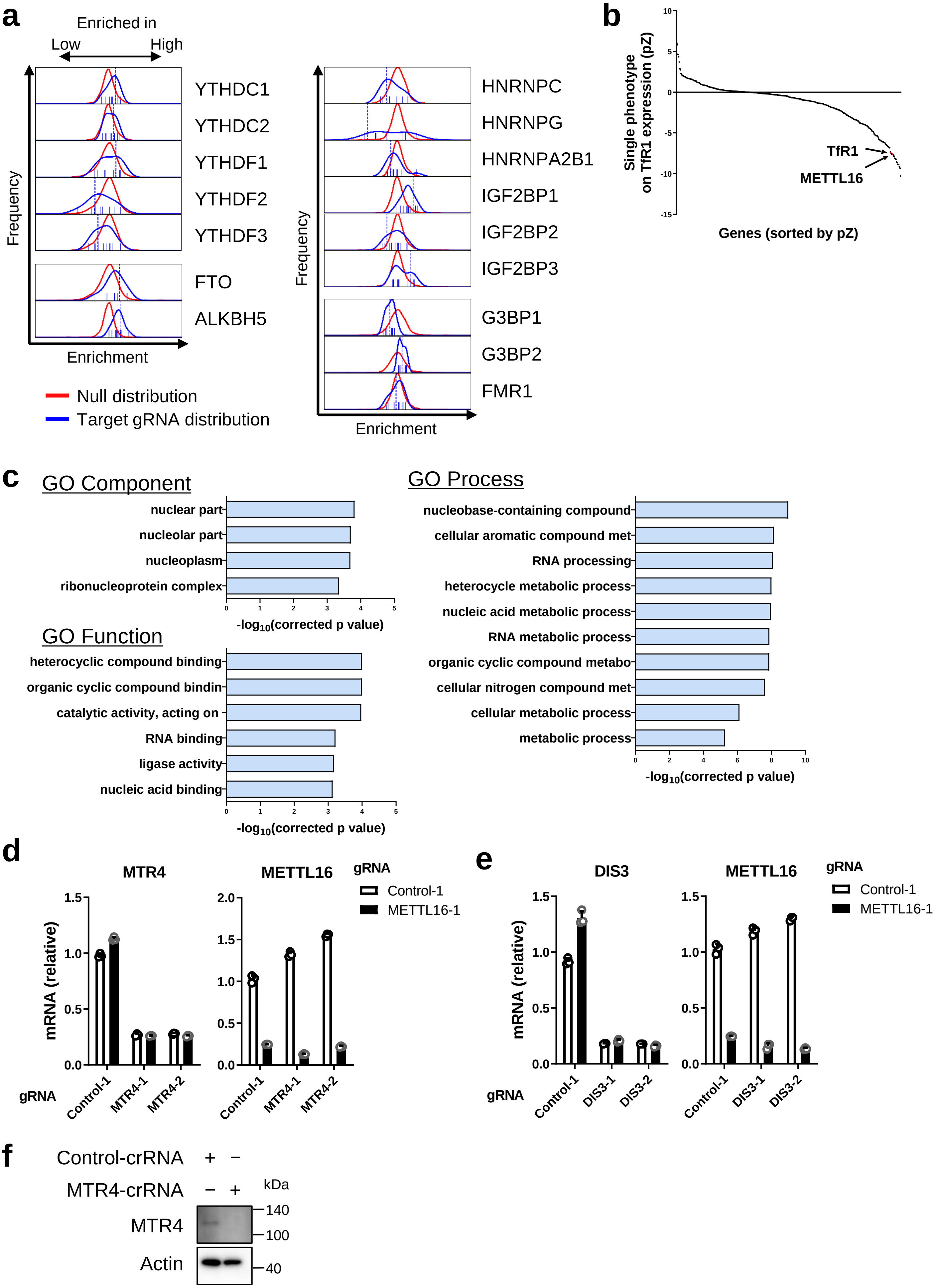
a
Enriched in
Low
High
Frequency
Enrichment
YTHDC1
YTHDC2
YTHDF1
YTHDF2
YTHDF3
FTO
ALKBH5
Null distribution
Target gRNA distribution
HNRNPC
HNRNPG
HNRNPA2B1
IGF2BP1
IGF2BP2
IGF2BP3
G3BP1
G3BP2
FMR1
b
Single phenotype on TfR1 expression (pZ)
Genes (sorted by pZ)
TfR1
METTL16
c
GO Component
nuclear part
nucleolar part
nucleoplasm
ribonucleoprotein complex
-log10(corrected p value)
GO Function
heterocyclic compound binding
organic cyclic compound bindin
catalytic activity, acting on
RNA binding
ligase activity
nucleic acid binding
-log10(corrected p value)
GO Process
nucleobase-containing compound
cellular aromatic compound met
RNA processing
heterocycle metabolic process
nucleic acid metabolic process
RNA metabolic process
organic cyclic compound metabo
cellular nitrogen compound met
cellular metabolic process
metabolic process
-log10(corrected p value)
d
MTR4
METTL16
mRNA (relative)
gRNA
Control-1
METTL16-1
Control-1
MTR4-1
MTR4-2
e
DIS3
METTL16
Control-1
DIS3-1
DIS3-2
f
Control-crRNA
MTR4-crRNA
kDa
MTR4
Actin
140
100
40

**Supplementary Fig. 12: Additional data regarding the METTL16 genetic interaction screen results.**

(a) Enrichment of gRNAs and estimated effect size for known $m^6A$-regulating proteins in the whole-genome TfR1 CRISPR screen performed in Fig. 1. Red lines represent the distribution of non-targeting controls. Blue vertical lines represent the enrichment of individual gRNAs targeting indicated genes. Blue curved lines represent the smoothed distribution of the enrichment of gRNAs targeting the indicated genes. Blue vertical dotted lines represent the effect size estimated by casTLE.

(b) Effects of single gene knockdown on TfR1 surface expression determined by the pairwise CRISPR screen.

(c) GO analysis of METTL16 buffering genes identified by the pairwise CRISPR screen. Statistical analysis was performed using Immuno-Navigator.

(d-e) Knockdown efficiency of the indicated gRNAs used in the validation experiments of pairwise CRISPR screen in Figs. 6e-h (n=3 technical replicates). Data are expressed as mean ± SD.

(f) Effects of the nucleofection of Cas9 and Mtr4-targeting crRNAs used in Fig. 6i.

Data are representative of at least two independent experiments (d-f). Source data are provided as a Source Data file.

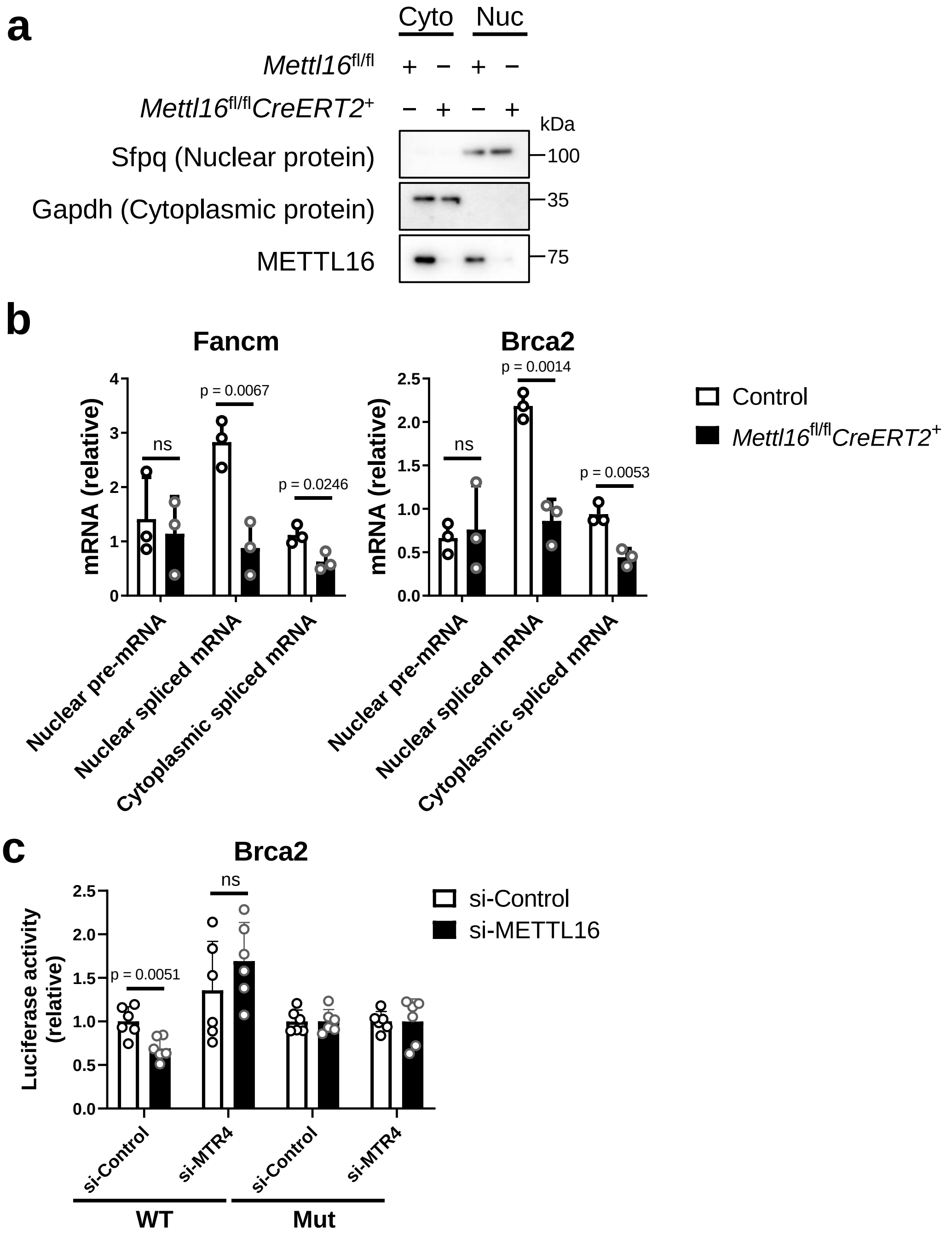


**Supplementary Fig. 13: Additional data regarding the nuclear RNA decay under METTL16 deficiency**
(a-b) Cytoplasmic and nuclear RNA fractionation. Cultured erythroblasts from control and *Mettl16*$^{fl/fl}$*CreERT2*$^{+}$ mice which were harvested, and subjected to the isolation of cytoplasmic and nuclear RNA fractions. (a) Confirmation of nuclear (nuc) and cytoplasmic (cyto) fractions by immunoblotting. (b) mRNA expression levels of indicated genes (pre-mRNAs and spliced mRNAs) in nuclear and cytoplasmic fractions (n=3 biological replicates). mRNA expression levels were normalized to the expression level of the corresponding pre-mRNA or spliced mRNA of β-actin (*Actb*). Similar results were obtained in at least two independent experiments.
(c) Firefly luciferase reporter activity of indicated reporters shown in Fig. 5f under the knockdown of METTL16, together with MTR4 in HeLa cells (n=6 biological replicates). Firefly luciferase activity values of the wild-type *Brca2* reporter were normalized to those of the mutant *Brca2* reporter, which lacks the METTL16 target motif (shown in Fig. 5f). Data were pooled from two independent experiments.
Data are expressed as mean ± SD (b, c). The p values were calculated using two-tailed Student's t test. ns, not significant. Source data are provided as a Source Data file.

| Name | Species | Gene | Type | Protospacer sequence |
|---|---|---|---|---|
| Control-gRNA1 | Human | Control | N/A | GAACCGTTGACGCGAAAA |
| Control-gRNA2 | Human | Control | N/A | GTCGTCCGGTTGGATCGA |
| hMETTL16-gRNA1 | Human | METTL16 | KO | GACGTGTACTCTCCTAA |
| hMETTL16-gRNA2 | Human | METTL16 | KO | GAGCTCTGACTGCTTCG |
| hFASN-gRNA1 | Human | FASN | KO | GAACTGCACGGAGGTGTT |
| hFASN-gRNA2 | Human | FASN | KO | GCCGGGCCAGGGACTTCT |
| hXPO5-gRNA1 | Human | XPO5 | KO | GGTAGCGCTGGGTGGAGTTG |
| hXPO5-gRNA2 | Human | XPO5 | KO | GTATCTGAAGAACAGTGTCA |
| hTfR1-gRNA1 | Human | TfR1 | KO | AAATTCATATGTCCCTCGTG |
| hTfR1-gRNA2 | Human | TfR1 | KO | TCTATAGTGATTGTCAGAGC |
| hIRP2-gRNA1 | Human | IRP2 | KO | GTTCTGCCTTACTCAATAC |
| hIRP2-gRNA2 | Human | IRP2 | KO | GCAGCAAAATCCACCATTGC |
| hFPN1-gRNA1 | Human | FPN1 | KO | GTGTTTCTGGTAGAGCTCTA |
| hFPN1-gRNA2 | Human | FPN1 | KO | GTACCACCAGCGAGGTCT |
| hTHOC6-gRNA1 | Human | THOC6 | KO | GCCGCCAGAAACTTCCCACA |
| hTHOC6-gRNA2 | Human | THOC6 | KO | GTGGAAACCATGCTATAGAC |
| hDMT1-gRNA1 | Human | DMT1 | KO | GCCACAGGATGACTCGT |
| hDMT1-gRNA2 | Human | DMT1 | KO | GAGAACGCCACCCCACAG |
| hFBXL5-gRNA1 | Human | FBXL5 | KO | GCACAACACTGCTCTCAGA |
| hFBXL5-gRNA2 | Human | FBXL5 | KO | GCTCTTCAGCATGATTCCAT |
| hSTEAP3-gRNA1 | Human | STEAP3 | KO | GGCTGTGCGTTTGGGGTTG |
| hSTEAP3-gRNA2 | Human | STEAP3 | KO | GTCCCGGACGAAGTTGT |
| hNCOA4-gRNA1 | Human | NCOA4 | KO | GTGTCAGCTTCAAAGAGC |
| hNCOA4-gRNA2 | Human | NCOA4 | KO | GTCTTAGAAGCCGTGAGGTA |
| hRC3H1-gRNA1 | Human | RC3H1 | KO | GAGGGCAGCTCGATCTTT |
| hRC3H1-gRNA2 | Human | RC3H1 | KO | GGGCAGCAGTAAGGGCTA |
| hHNRNPR-gRNA1 | Human | HNRNPR | KO | GTTCTGCTTCCTTGAATATG |
| hHNRNPR-gRNA2 | Human | HNRNPR | KO | GATGACAAAAAGAAGAATCG |
| hHNRNPL-gRNA1 | Human | HNRNPL | KO | GGTGACCAGCAATGTATATT |
| hHNRNPL-gRNA2 | Human | HNRNPL | KO | GTCAATCAGGCCCCTGATG |
| hMETTL16-gRNAi1 | Human | METTL16 | CRISPRi | GTTTCTAGGAGCTTCGCTATG |
| hMETTL16-gRNAi2 | Human | METTL16 | CRISPRi | GAGCGTATCATCTGCGTTTCT |
| hMTR4-gRNAi1 | Human | MTR4 | CRISPRi | GCGGGGCATCGTGGGTAGGA |
| hMTR4-gRNAi2 | Human | MTR4 | CRISPRi | GGAGGGCGACTCGACCACTG |
| hDIS3-gRNAi1 | Human | DIS3 | CRISPRi | GTCTAGAATACGCCTAACCC |
| hDIS3-gRNAi2 | Human | DIS3 | CRISPRi | GAGAATACGCCTAACCCCGG |
| mMtr4-crRNA1 | Mouse | Mtr4 | KO | CATCTGGATGTACTCGCCAG |
| mMtr4-crRNA2 | Mouse | Mtr4 | KO | ATGAAGAATTTCAAGATGTG |
| mMettl3-crRNA | Mouse | Mettl3 | KO | GAGTTGATTGAGGTAAAGCG |
| mBrca2-crRNA | Mouse | Brca2 | KO | ATCTGATTTCATATTCAAGG |
| mFancm-crRNA | Mouse | Fancm | KO | GGTGATATGGCTCTACCCAG |

Supplementary Table 1: List of Protospacer sequences

| Species | Gene | Forward primer | Reverse primer |
|---|---|---|---|
| Human | ACTB | CACCATTGGCAATGAGCGGTTCC | CTTCTGCATCCTGTCGGCAATGC |
| Human | METTL16 | TGGCTGGTATTTCCTCGCAA | GGAGTGTCTTCTGTGGCACT |
| Human | TfR1 | CTGCCAGCCCACTGTTGTAT | GCCCAGTTGCTGTCCTGATA |
| Human | HBG | AGACGCCATGGGTCATTTCA | GTAGACAACCAGGAGCCTTCC |
| Human | MTR4 | GGACAAGGGGAAATGGAAGGG | AAATGGGTTCATCTGTACCTTCG |
| Human | DIS3 | TGGAGCTTCTCCCCAAGAGT | AGGCTTCAACATTTTCTCGCT |
| Mouse | Actb | GGCTGTATTCCCCTCCATCG | CCAGTTGGTAACAATGCCATGT |
| Mouse | Gapdh | AGGTCGGTGTGAACGGATTTG | TGTAGACCATGTAGTTGAGGTCA |
| Mouse | Mettl16 | TTGGTCACCAGGATTCGGAC | TGCCAGGAAATACCAGCCAT |
| Mouse | Mat2a | GCGTTCATTGAGGAGGGGAC | GCCACTTTAGCATCAGGGTC |
| Mouse | TfR1 | GCTAGTGTCAGAAAACCCAAGAGG | GTTTCAGCCAGTTTCACACACTCC |
| Mouse | Alas2 | GGGCTAAGAGCCATTGTCCT | ATGGCTTCGGGTGGTTGAAT |
| Mouse | Hba | CCGTCAACTTCAAGCTCCTG | TGCTCACAGAGGCAAGGAAT |
| Mouse | Gypa | CAGCCTGTCTCACCACACAT | ATAATCCCTGCCATCACGCC |
| Mouse | Slc4a1 | TTTGAAGGCAGCTTCCTCGT | ACAGGGGCATAAGTCTGTTGT |
| Mouse | Epb41 | GTTGAAGAGAAGCGGGGAGAA | TAGGTACTGTGACCTCGGTGT |
| Mouse | Steap3 | GCTCCCGTCCATTGCTAATTC | GTGTGTGCATTGTGCTGAGT |
| Mouse | Fech | CATCATGCCAAGACCACCAA | TGTCATGAGGTCTCGGTCCAG |
| Mouse | Gata2 | CTCCAGCTTCACCCCTAAGC | AGGCATTGCACAGGTAGTGG |
| Mouse | Gata1 | TAAGGTGGCTGAATCCTCTGC | CAGAATCCACAAACTGGGGC |
| Mouse | Klf1 | CTACACCAAGAGCTCGCACC | AAGGGACGATGTCCAGTGTG |
| Mouse | Epor | GCTTCCTGAAGCTAGGGCTG | CAGGGCCGCTTTGCTCTCA |
| Mouse | Jak2 | GGCGACGGGAACAAGATGTG | CTGTCATTGTAAGGCAGGCCA |
| Mouse | Myc | CTCGAGCTGTTTGAAGGCTG | AATAGGGCTGTACGGAGTCG |
| Mouse | Spi1 | GCCCCTCCATCGGATGAC | CATGGTGTGCGGAGAAATCC |
| Mouse | Cebpb | GCAATCCGGATCAAACGTGG | GATTACTCAGGGCCCGGCTG |
| Mouse | Nfkbia | AGCTCCGAGACTTTCGAGGA | CACGTGTGGCCATTGTAGTTG |
| Mouse | Nfkb2 | CTTGTCTGACATGGGTCTCCA | ACTGTCTTCTTTCACCTCTGTGCT |
| Mouse | Hmox1 | CAGAAGAGGCTAAGACCGCC | ATCTTGCACCAGGCTAGCAG |
| Mouse | Mpo | TTCGAGAGCATAACCGGCTG | GCAGGTAGTCCCGGTATGTG |
| Mouse | Lyz2 | GGCAAAACCCCAAGAGCTGTG | TGGGGATCTCTCACCACCCT |
| Mouse | Brca1 | GATCCAGCACCTCTCTTGGG | ACTTCTTGAATTTGGACGGCAG |
| Mouse | Brca2 | CAAAACTCAAGGAGGGTCAGACA | GCGTGCTTCTTCATCTCGGG |
| Mouse | Fancm | AGCACCGGCCAGTTATTAGAG | TAGCCACCGTCTCGGAACT |
| Mouse | Fancf | AACTCCTGGACACGTTGTGG | TTCTGTGTCTCATCCGCACC |
| Mouse | Rfwd3 | GACCAGGAATCCACCGTCAG | AGCTTCTTGGTCTGTTGCGA |
| Mouse | Rmi2 | GACTCGAGCGGAGCCTTCTC | TCACCATCACATACTTTCCTGGG |
| Mouse | Rad54b | CCAAGTCAGGTGCAGGGAAAGT | ACACCTGGATCATTTTGTGACTGT |
| Mouse | Blm | AGGACCTGCTGGAAGATTTACTC | GTCTTTCGGCATTATGTCGTG |
| Mouse | Pole | GGTGCGTCCTACCCAATAGC | TCCTGGTACTGGTGGTTGGT |
| Mouse | U4 snRNA | GCAGTGGCAGTATCGTAGCC | AAAAATTGCCAGTGCCGACT |
| Mouse | U6 snRNA | GCTCGCTTCGGCAGCACATAT | ATATGGAACGCTTCACGAATTTGC |
| Mouse | pre-Brca2 | ACAAGCTTCTGCGTGATTGC | GGATCCACACCTGGAGTGTC |
| Mouse | pre-Fancm | CCAGTACCGGGATCTAGGCT | CAACTGAGCGCACCAATGTG |
| Mouse | pre-Actb | AGGACAGGGGCTCCACTTAG | TTCTGCTCTTTCCCAGACGAG |
| Common | SELECT | ATGCAGCGACTCAGCCTCTG | TAGCCAGTACCGTAGTGCGTG |

Supplementary Table 2: List of qPCR primers

| Species | Gene | Site | Primer 1 (up) | Primer 2 (down) |
|---|---|---|---|---|
| Mouse | Mat2a | A2694 | tagccagtaccgtagtgcgtgCTGAAAGGCACTTTCTTGCT | 5phos/AGGGCAAGCAGTCATGGAAcagaggctgagtcgctgcat |
| Mouse | Mat2a | A1351 | tagccagtaccgtagtgcgtgGAGCTTGAAGGCTTCTC | 5phos/GTAACCTACGCCAACAAGcagaggctgagtcgctgcat |
| Mouse | Brca1 | A2375 | tagccagtaccgtagtgcgtgGGCGTGTTTCAAGTTGCTC | 5phos/GTTCCTGTTCTCTGAGGGCTcagaggctgagtcgctgcat |
| Mouse | Fancf | A852 | tagccagtaccgtagtgcgtgCCAGGGCATGTCCTCCATC | 5phos/GTGTAGGGACCCAGGCACcagaggctgagtcgctgcat |
| Mouse | Brca2 | A10210 | tagccagtaccgtagtgcgtgCTACTTGCTGGTCTGGCCTC | 5phos/GTGCTGGCTGGGGGTTCCTTcagaggctgagtcgctgcat |
| Mouse | Fancm | A6958 | tagccagtaccgtagtgcgtgCATCAAAGATGACTCATCTTTTC | 5phos/GTGTATGAATGAAGATGTGAACAcagaggctgagtcgctgcat |

Supplementary Table 3: List of SELECT primers

| Name | Type | Sequence |
|---|---|---|
| Mettl16L1 | Protospacer | GGTATCATTGAGAAGTTGTA |
| Mettl16L1 | Donor oligo | TT*C*C*CTAGATTTGTAACTCTGGATTTTCACAACTGGGCTGCAGGTATCATTGAGAAGTTGAAGCTTATAACTTCGTATAATGTATGCTATACGAAGTTATTAAGGCAGTAATTAAGTTACTGTTTATGCTCTTGAGAAAATGCTTTCAAACTTTGTT*T*T*G |
| Mettl16R4 | Protospacer | CCCAACCAACACAGATGCTA |
| Mettl16R4 | Donor oligo | TG*T*T*GTTTAATTAAAAGTTGTTTAATAACAGAGTAGACAATGCCCAACCAACACAGATGCATAACTTCGTATAGCATACATTATACGAAGTTATACTAGTTAAGGTAAAAAGTTGAAAGCTAAGTAAACCAGGACCTTTTCTTCCTTCCACGCTTCT*C*T*A |

*Phosphorothioate bond modification

Supplementary Table 4: List of oligonucleotides used for the generation of floxed mice

| Species | Gene | Sense strand | Antisense strand |
|---|---|---|---|
| Human | METTL16 | GGAAUUAUCCCUCAAAGCAtt | UGCUUUGAGGGAUAAUUCCtt |
| Human | MTR4 | GCUUUACAGAGGUUCCGAAtt | UUCGGAACCUCUGUAAAGCat |

Supplementary Table 5: List of siRNA sequences

# Supplementary References

1. Supek, F., Bosnjak, M., Skunca, N., and Smuc, T. (2011). REVIGO summarizes and visualizes long lists of gene ontology terms. PloS one *6*, e21800.
2. Vitting-Seerup, K., and Sandelin, A. (2019). IsoformSwitchAnalyzeR: analysis of changes in genome-wide patterns of alternative splicing and its functional consequences. Bioinformatics *35*, 4469-4471.
3. Kuppers, D.A., Arora, S., Lim, Y., Lim, A.R., Carter, L.M., Corrin, P.D., Plaisier, C.L., Basom, R., Delrow, J.J., Wang, S., *et al.* (2019). N(6)-methyladenosine mRNA marking promotes selective translation of regulons required for human erythropoiesis. Nature communications *10*, 4596.
