# Supplementary material for: The N6-methyladenosine methyltransferase METTL16 enables erythropoiesis through safeguarding genome integrity
Source: Nat Commun. 2022 Oct 28;13:6435. doi: 10.1038/s41467-022-34078-y (PMC9616860; doi:10.1038/s41467-022-34078-y)
Supplement: Supplementary file 3 — Description of Additional Supplementary Files [file 41467_2022_34078_MOESM3_ESM.pdf]

Description of Additional Supplementary Files

Supplementary Data 1: Results of the CRISPR screens to identify TfR1 regulators in K562 cells.

Supplementary Data 2: Transcriptome analysis comparing $CD71^{+}Ter119^{–}$ erythroblasts from control and *Mettl16*$^{fl/fl}$*Epor-Cre*$^{+}$ mice.

Supplementary Data 3: Transcriptome analysis comparing $CD71^{+}Ter119^{+}$ erythroblasts from control and *Mettl16*$^{fl/fl}$*Epor-Cre*$^{+}$ mice.

Supplementary Data 4: MeRIP-seq analysis comparing cultured erythroblasts from control and *Mettl16*$^{fl/fl}$*CreERT2*$^{+}$ mice.

Supplementary Data 5: Transcriptome analysis comparing cultured erythroblasts from control and *Mettl16*$^{fl/fl}$*CreERT2*$^{+}$ mice.

Supplementary Data 6: Results of the pairwise CRISPR screen to identify METTL16 genetic interactors in K562 cells.
