# Supplementary material for: The N6-methyladenosine methyltransferase METTL16 enables erythropoiesis through safeguarding genome integrity
Source: Nat Commun. 2022 Oct 28;13:6435. doi: 10.1038/s41467-022-34078-y (PMC9616860; doi:10.1038/s41467-022-34078-y)
Supplement: Supplementary file 10 — Reporting Summary [file 41467_2022_34078_MOESM10_ESM.pdf]

nature portfolio

Corresponding author(s): Osamu Takeuchi
Masanori Yoshinaga

Last updated by author(s): Oct 6, 2022

Reporting Summary

Nature Portfolio wishes to improve the reproducibility of the work that we publish. This form provides structure for consistency and transparency in reporting. For further information on Nature Portfolio policies, see our Editorial Policies and the Editorial Policy Checklist.

Statistics

For all statistical analyses, confirm that the following items are present in the figure legend, table legend, main text, or Methods section.

n/a	Confirmed	
☐	☒	The exact sample size (*n*) for each experimental group/condition, given as a discrete number and unit of measurement
☐	☒	A statement on whether measurements were taken from distinct samples or whether the same sample was measured repeatedly
☐	☒	The statistical test(s) used AND whether they are one- or two-sided<br>*Only common tests should be described solely by name; describe more complex techniques in the Methods section.*
☒	☐	A description of all covariates tested
☐	☒	A description of any assumptions or corrections, such as tests of normality and adjustment for multiple comparisons
☐	☒	A full description of the statistical parameters including central tendency (e.g. means) or other basic estimates (e.g. regression coefficient) AND variation (e.g. standard deviation) or associated estimates of uncertainty (e.g. confidence intervals)
☐	☒	For null hypothesis testing, the test statistic (e.g. *F*, *t*, *r*) with confidence intervals, effect sizes, degrees of freedom and *P* value noted<br>*Give P values as exact values whenever suitable.*
☒	☐	For Bayesian analysis, information on the choice of priors and Markov chain Monte Carlo settings
☒	☐	For hierarchical and complex designs, identification of the appropriate level for tests and full reporting of outcomes
☒	☐	Estimates of effect sizes (e.g. Cohen's *d*, Pearson's *r*), indicating how they were calculated

Our web collection on statistics for biologists contains articles on many of the points above.

Software and code

Policy information about availability of computer code

Data collection	Flow cytometric data were collected using FACSVerse (BD Biosciences), LSRFortessa X-20 (BD Biosciences) and SH800Z (Sony).
Data analysis	The following softwares and scripts were used for analysis: Python (v2.7, or v3.7), casTLE (v0.7), IsoformSwitchAnalyzeR (v1.5.11), cutadapt (v2.3), Trim_Galore (v0.6.4), hisat2 (v2.1.0), R (v3.6 or v4.1), featureCounts (v1.6.5), limma (v3.47.15), RADAR (v0.2.1), MEME (v5.4.1), mfold (v4.7), FlowJo (v7.6.5), CASPLab (v1.2.2) and Prism (v8.4.3). No custom codes were generated in this study.

For manuscripts utilizing custom algorithms or software that are central to the research but not yet described in published literature, software must be made available to editors and reviewers. We strongly encourage code deposition in a community repository (e.g. GitHub). See the Nature Portfolio guidelines for submitting code & software for further information.

Data

Policy information about availability of data

All manuscripts must include a data availability statement. This statement should provide the following information, where applicable:

- Accession codes, unique identifiers, or web links for publicly available datasets
- A description of any restrictions on data availability
- For clinical datasets or third party data, please ensure that the statement adheres to our policy

The raw sequencing data generated in this study have been deposited in the DDBJ Sequence Read Archive under accession code DRA013293 [https://ddbj.nig.ac.jp/resource/sra-submission/DRA013293], and DRA013294 [https://ddbj.nig.ac.jp/resource/sra-submission/DRA013294]. The processed data reported in this paper are tabulated in the Supplementary Data sets. Source data underlying all graphs generated in this study are provided in the Supplementary Information/Source Data file. All data needed to evaluate the conclusions in the study are present in the article and/or Supplementary Information. The Mus musculus mouse (GRCm38/mm10) reference genome and annotations used in this study is available from the GENCODE [https://www.gencodegenes.org/mouse/release_M10.html] or UCSC

[https://hgdownload.soe.ucsc.edu/goldenPath/mm10/] server. The processed RNA-seq data published by Kuppers et al. are available in the NCBI Gene Expression Omnibus under accession number GSE106124 [https://www.ncbi.nlm.nih.gov/geo/query/acc.cgi?acc=GSE106124]. Source data are provided with this paper.

Field-specific reporting

Please select the one below that is the best fit for your research. If you are not sure, read the appropriate sections before making your selection.

- [x] Life sciences
- [ ] Behavioural & social sciences
- [ ] Ecological, evolutionary & environmental sciences

For a reference copy of the document with all sections, see nature.com/documents/nr-reporting-summary-flat.pdf

Life sciences study design

All studies must disclose on these points even when the disclosure is negative.

Sample size	No statistical methods were used to predetermine the sample size. Sample sizes were determined based on our previous experience (PMID: 28538180) or pilot experiments.
Data exclusions	No data were excluded from analyses.
Replication	Mouse and in vitro experiments were conducted and successfully replicated with sufficient numbers of mice or biological replicates as indicated in the Figure Legends section. All the replicates in NGS analyses showed consistent reproducibility. The genetic interaction screen was done once with sufficient numbers of gRNA pairs (at least 35 combinations for each gene pair) and further validated using individiual gRNA sets for at least three times.
Randomization	Randomization was not required in this study because there is no statistic that requires randomization of samples. For all animal experiments, control or knock-out mice were randomly selected from the littermates. For other experiments, all samples were treated in the same way to decrease the variability.
Blinding	The experiments were not performed blindly due to the researchers needed to verify the genotypes of cell lines or mice.

Reporting for specific materials, systems and methods

We require information from authors about some types of materials, experimental systems and methods used in many studies. Here, indicate whether each material, system or method listed is relevant to your study. If you are not sure if a list item applies to your research, read the appropriate section before selecting a response.

Materials & experimental systems

n/a	Involved in the study	
[ ]	[x]	Antibodies
[ ]	[x]	Eukaryotic cell lines
[x]	[ ]	Palaeontology and archaeology
[ ]	[x]	Animals and other organisms
[x]	[ ]	Human research participants
[x]	[ ]	Clinical data
[x]	[ ]	Dual use research of concern

Methods

n/a	Involved in the study	
[x]	[ ]	ChIP-seq
[ ]	[x]	Flow cytometry
[x]	[ ]	MRI-based neuroimaging

Antibodies

Antibodies used	Primary antibodies for immunoblot and immunofluorescence analysis used in this study were as follows: anti-METTL16 (Bethyl Laboratorties, #A304-192A, 1:1000), anti-transferrin receptor (Santa Cruz Biotechnology, #sc-32272, 1:1000), anti-β actin (Santa Cruz Biotechnology, #sc-1615, 1:5000), anti-Phospho-Histone H2A.X (Ser139, Cell Signaling Technology, #2577, 1:800), anti-cleaved caspase-3 (Cell Signaling Technology, #9664, 1:2000), anti-GATA1 (Cell Signaling Technology, #3535, 1:500), anti-FASN (Santa Cruz Biotechnology, #sc-55580, 1:1000), anti-XPO5 (Santa Cruz Biotechnology, #sc-271036, 1:1000), anti-HNRNPL (Santa Cruz Biotechnology, #sc-32317, 1:1000), anti-MTR4 (Santa Cruz Biotechnology, #sc-515828, 1:1000), anti-METTL3 (Proteintech, #15073-1-AP, 1:1000), anti-BRCA2 (Abcam, #ab27976, 1:1000), anti-FANCM (Bethyl Laboratorties, #A302-637A, 1:1000), anti-GAPDH (Santa Cruz Biotechnology, #sc-47724, 1:1000), anti-SFPQ (MBL, #RN014MW, 1:1000) and anti-FLAG (Sigma Aldrich, #F3165, 1:5000). Secondary HRP-conjugated antibodies were from Cytiva (Anti-Mouse IgG, HRP-Linked F(ab')2 Fragment Sheep, #NA9310, and Anti-Rabbit IgG, HRP-Linked F(ab')2 Fragment Donkey, #NA9340, 1:5000).<br>Antibodies and fluorescent dyes for flow cytometry analysis were as follows: Brilliant Violet 421 Rat anti-mouse CD16/32 (Clone: 93, Biolegend, #101332, 1:200), FITC Mouse anti-human CD71 (Clone: CY1G4, Biolegend, #334104, 1:100), FITC Rat anti-mouse CD34 (Clone: RAM34, BD Pharmingen, #560238, 1:100), PerCP/Cy5.5 Streptavidin (Biolegend, #405214, 1:500), PE Rat anti-mouse Ter119 (Clone: TER-119, Biolegend, #116207, 1:200), PE Rat anti-mouse c-kit (Clone: 2B8, Biolegend, #105808, 1:200), PE/Cy7 Rat anti-mouse Ki67 (Clone: 16A8, Biolegend, #652425, 1:200), PE/Cy7 Annexin V (Biolegend, #640949, 1:200), PE/Cy7 Rat anti-mouse Sca-1 (Clone: D7, Biolegend, #108114, 1:200), APC Mouse anti-human CD71 (Clone: CY1G4, Biolegend, #334107, 1:200), and APC Rat anti-mouse CD71 (Clone: RI7217, Biolegend, #113819, 1:200).

Validation	The validities of anti-METTL16, anti-FASN, anti-XPO5, anti-HNRNPL, and anti-MTR4 antibodies were confirmed using knockout cell lines (Supplementary Figs. 1f, 12f). All other antibodies were validated for the indicated applications by well-known suppliers as follows:<br>anti-transferrin receptor (Santa Cruz Biotechnology, #sc-32272):https://www.scbt.com/p/cd71-antibody-3b8-2a1<br>anti-β actin (Santa Cruz Biotechnology, #sc-1615):https://www.scbt.com/ja/p/actin-antibody-c-11<br>anti-Phospho-Histone H2A.X (Ser139, Cell Signaling Technology, #2577):https://www.cellsignal.jp/products/primary-antibodies/phospho-histone-h2a-x-ser139-antibody/2577<br>anti-cleaved caspase-3 (Cell Signaling Technology, #9664):https://www.cellsignal.jp/products/primary-antibodies/cleaved-caspase-3-asp175-5a1e-rabbit-mab/9664<br>anti-GATA1 (Cell Signaling Technology, #3535):https://www.cellsignal.jp/products/primary-antibodies/gata-1-d52h6-xp-rabbit-mab/3535<br>anti-XPO5 (Santa Cruz Biotechnology, #sc-271036):https://www.scbt.com/ja/p/exportin-5-antibody-a-11<br>anti-METTL3 (Proteintech, #15073-1-AP):https://www.ptglab.co.jp/products/METTL3-Antibody-15073-1-AP.htm<br>anti-BRCA2 (Abcam, #ab27976):https://www.abcam.co.jp/brca2-antibody-ab27976.html<br>anti-FANCM (Bethyl Laboratorties, #A302-637A):https://www.fortislife.com/products/primary-antibodies/rabbit-anti-fancm-antibody/BETHYL-A302-637<br>anti-GAPDH (Santa Cruz Biotechnology, #sc-47724):https://www.scbt.com/ja/p/gapdh-antibody-0411<br>anti-SFPQ (MBL, #RN014MW):https://ruo.mbl.co.jp/bio/dtl/A/?pcd=RN014MW<br>anti-FLAG (Sigma Aldrich, #F3165):https://www.sigmaaldrich.com/JP/ja/product/sigma/f3165<br>Brilliant Violet 421 Rat anti-mouse CD16/32 (Clone: 93, Biolegend, #101332):https://www.biolegend.com/ja-jp/products/brilliant-violet-421-anti-mouse-cd16-32-antibody-8598<br>FITC Mouse anti-human CD71 (Clone: CY1G4, Biolegend, #334104):https://www.biolegend.com/ja-jp/products/fitc-anti-human-cd71-antibody-4907<br>FITC Rat anti-mouse CD34 (Clone: RAM34, BD Pharmingen, #560238):https://www.bdbiosciences.com/en-au/products/reagents/flow-cytometry-reagents/research-reagents/single-color-antibodies-ruo/fitc-rat-anti-mouse-cd34.560238<br>PerCP/Cy5.5 Streptavidin (Biolegend, #405214):https://www.biolegend.com/ja-jp/products/percp-cyanine5-5-streptavidin-4212<br>PE Rat anti-mouse Ter119 (Clone: TER-119, Biolegend, #116207):https://www.biolegend.com/ja-jp/products/pe-anti-mouse-ter-119-erythroid-cells-antibody-1867<br>PE Rat anti-mouse c-kit (Clone: 2B8, Biolegend, #105808):https://www.biolegend.com/ja-jp/products/pe-anti-mouse-cd117-c-kit-antibody-75<br>PE/Cy7 Rat anti-mouse Ki67 (Clone: 16A8, Biolegend, #652425):https://www.biolegend.com/ja-jp/products/pe-cyanine7-anti-mouse-ki-67-antibody-13821?GroupID=GROUP26<br>PE/Cy7 Annexin V (Biolegend, #640949):https://www.biolegend.com/ja-jp/products/pecyanine7-annexin-v-14931<br>PE/Cy7 Rat anti-mouse Sca-1 (Clone: D7, Biolegend, #108114):https://www.biolegend.com/ja-jp/products/pe-cyanine7-anti-mouse-ly-6a-e-sca-1-antibody-3137<br>APC Mouse anti-human CD71 (Clone: CY1G4, Biolegend, #334107):https://www.biolegend.com/ja-jp/products/apc-anti-human-cd71-antibody-7517<br>APC Rat anti-mouse CD71 (Clone: RI7217, Biolegend, #113819):https://www.biolegend.com/ja-jp/products/apc-anti-mouse-cd71-antibody-15498

Eukaryotic cell lines

Policy information about cell lines

Cell line source(s)	HEK293T (ATCC, #CRL-3216), K562 (ATCC, #CCL-243), HeLa (ATCC, #CCL-2), NIH3T3 (ATCC, #CRL-1658)
Authentication	Additional authentication was not performed in this study.
Mycoplasma contamination	All cell lines were mycoplasma negative.
Commonly misidentified lines (See ICLAC register)	No commonly misidentified line was used in this study.

Animals and other organisms

Policy information about studies involving animals; ARRIVE guidelines recommended for reporting animal research

Laboratory animals	Mice of both sexes (Mettl16-floxed, Ifnar1-deficient, Tmem173-gt, Epor-Cre, ROSA26-CreERT2) were used on the indicated embryonic days (E10.5-13.5). C57BL/6JJcl or BDF1 strains were used. The strains used in this study were described in the Methods section. Mice were maintained at Kyoto University animal facilities with a 12 h light/dark cycle and access to food and water ad libitum. Room temperature was maintained at 23 ± 3 °C, with a relative humidity of 50 ± 20%.
Wild animals	This study does not involve wild animals.
Field-collected samples	This study does not involve field-collected samples.
Ethics oversight	All animal experiments were conducted in compliance with the regulations approved by the Committee for Animal Experiments of the Institute for Frontier Life and Medical Sciences and Graduate School of Medicine, Kyoto University.

Note that full information on the approval of the study protocol must also be provided in the manuscript.

Flow Cytometry

Plots

Confirm that:

- [x] The axis labels state the marker and fluorochrome used (e.g. CD4-FITC).
- [x] The axis scales are clearly visible. Include numbers along axes only for bottom left plot of group (a 'group' is an analysis of identical markers).
- [x] All plots are contour plots with outliers or pseudocolor plots.
- [x] A numerical value for number of cells or percentage (with statistics) is provided.

Methodology

Sample preparation	Mouse fetal livers or bone marrow cells were collected and single cell suspension was prepared following standard procedures. The detail of the procedures of sample preparation are described in the Methods section.
Instrument	FACSVerse (BD Biosciences), LSRFortessa X-20 (BD Biosciences) and SH800Z (Sony).
Software	FlowJo (v7.6.5, BD Biosciences).
Cell population abundance	For erythroblast isolation experiments, the purity of post-sort samples was >90%, which was determined by May-Grunwald-Giemsa staining in the pilot experiment.
Gating strategy	For CRISPR screens, cells were selected based on FSC/SSC, and then mCherry positive cells were selected as lentivirus-transduced cells. For animal experiments, fetal liver cells or bone marrow cells were selected based on FSC/SSC, and then Zombie/FVD positive cells were excluded as dead cells. Unstained or untransduced cells were used to determine the boundaries between negative and positive populations. Further gating strategies were shown in Figs. 2H and 2K.

- [x] Tick this box to confirm that a figure exemplifying the gating strategy is provided in the Supplementary Information.
